# Genetically encoding thioacetyl‐lysine as a non‐deacetylatable analog of lysine acetylation in *Escherichia coli*

**DOI:** 10.1002/2211-5463.12320

**Published:** 2017-10-16

**Authors:** Sumana Venkat, Dharma Theja Nannapaneni, Caroline Gregory, Qinglei Gan, Matt McIntosh, Chenguang Fan

**Affiliations:** ^1^ Department of Chemistry and Biochemistry University of Arkansas Fayetteville AR USA; ^2^ Department of Biological Sciences University of Arkansas Fayetteville AR USA

**Keywords:** deacetylation, genetic code expansion, non‐canonical amino acid, protein acetylation, synthetic biology, thioacetyl‐lysine

## Abstract

Reversible lysine acetylation is one of the most widely distributed post‐translational modifications; it is involved in a variety of biological processes and can be found in all three domains of life. Acetyltransferases and deacetylases work coordinately to control levels of protein acetylation. In this work, we applied the genetic code expansion strategy to site‐specifically incorporate *N* ^ε^‐thioacetyl‐l‐lysine (TAcK) as an analog of *N* ^ε^‐acetyl‐l‐lysine (AcK) into green fluorescent protein and malate dehydrogenase in *Escherichia coli*. We showed that TAcK could serve as an ideal functional mimic for AcK. It could also resist the bacterial sirtuin‐type deacetylase CobB. Thus, genetic incorporation of TAcK as a non‐deacetylatable analog of AcK into proteins will facilitate *in vivo* studies of protein acetylation.

**Abbreviations**
AcK: *N* ^ε^‐acetyl‐l‐lysine
AcKRS: acetyllysyl‐tRNA synthetase
MDH: malate dehydrogenase
MS: mass spectrometry
ncAA: non‐canonical amino acid
PylRS: pyrrolysyl‐tRNA synthetase
sfGFP: superfolder green fluorescent protein
SOC: Super Optimal broth with Catabolite repression
TAcK: *N* ^ε^‐thioacetyl‐l‐lysine
TAcKRS: thioacetyllysyl‐tRNA synthetase

The reversible acetylation of lysine residues in proteins has been recognized as one of the most widely distributed post‐translational modifications in both eukaryotes and prokaryotes 1, 2, 3, 4, 5. It plays key roles in regulating a wide range of important biological processes including gene transcription, stress response, apoptosis, cellular differentiation and metabolism 6, 7, 8, 9, 10, 11, 12, 13. Accumulating evidence has shown that protein acetylation is tightly associated with many human diseases such as cancers, cardiovascular diseases, diabetes and neurodegenerative disorders 14, 15, 16, 17, 18, 19, 20. Studies on protein acetylation have not only deepened our knowledge of its functions and mechanisms but also guided therapeutic strategies for acetylation‐associated diseases 21, 22, 23, 24.

Lysine acetylation can take place with or without acetyltransferases, while the removal of the acetyl group is mediated by deacetylases 25, 26, 27, 28, 29, 30, 31, 32, 33. Due to the existence of acetyltransferases and deacetylases as well as non‐enzymatic acetylation in cells, one challenge for studying lysine acetylation is to synthesize homogeneously acetylated proteins at specific sites. To overcome this problem, the genetic code expansion strategy was applied to utilize an orthogonal pair of an evolved pyrrolysyl‐tRNA synthetase (PylRS) variant and its cognate tRNA from *Methanosarcinaceae* species to cotranslationally incorporate *N* ^ε^‐acetyl‐l‐lysine (AcK) in response to a stop codon at the desired position in target proteins 34, 35, 36. This approach has already proved to be a powerful tool to study protein acetylation 37, 38, 39, 40, 41, 42, 43, 44, 45.

As a non‐hydrolyzable analog of AcK, *N* ^ε^‐thioacetyl‐l‐lysine (TAcK) has been used as an inhibitor for a number of deacetylases in the form of peptide or non‐peptide substrates 46, 47, 48, 49, 50, 51, 52. Recently, Söll's group utilized the flexizyme‐mediated tRNA aminoacylation and cell‐free translation systems to incorporate TAcK into histone H3, which was shown to be resistant to SIRT1 deacetylase 53. However, this approach can only be used *in vitro*. In this work, we established an *in vivo* system to site‐specifically incorporate TAcK into proteins for broader applications by using living cells.

## Materials and methods

### Thioacetyl‐lysine synthesis

**Figure 1:**
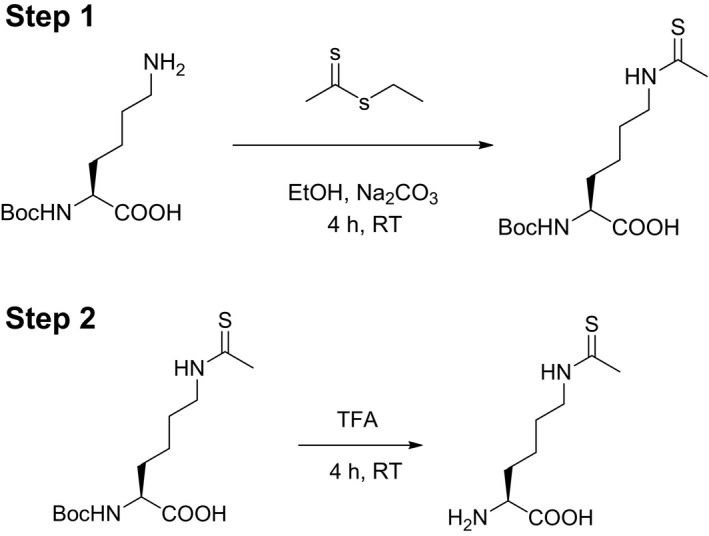
The Scheme for TAcK synthesis.

The synthesis of TAcK followed a previous protocol with slight modifications (Fig. 1) 53.

#### Step 1

A 5% (w/v) aqueous solution of Na_2_CO_3_ (25 mL) was added dropwise to a stirred suspension of *N* ^α^‐Boc‐l‐lysine (3.0 g, 12 mmol) in ethanol (25 mL) at 0 °C. Ethyl dithioacetate (1.5 mL, 13.2 mmol) was then added dropwise at 0 °C. After the addition was complete, the reaction mixture was stirred at room temperature for 4 h before the addition of a 50% (v/v) solution of ethanol in water (3 mL). The ethanol was removed under reduced pressure and the residue was acidified with 6 m HCl to pH ~ 1–2 and extracted with CH_2_Cl_2_. The combined organic extracts were washed with saturated NaCl solution, dried over anhydrous Na_2_SO_4_, filtered and evaporated to dryness, affording an oily residue. The product was isolated as a white solid (3.0 g, 68%) after silica gel column chromatography (60 : 40 ethyl acetate/hexanes). Spectral data of the compound match with the previous report 53: ^1^H‐NMR (400 MHz, CDCl_3_) δ: 8.61 (s, 1H), 4.16 (m, 1H), 3.49–3.55 (m, 2H), 2.46 (s, 3H), 1.62–1.78 (m, 6H), 1.37 (s, 9H) (Fig. S1); ^13^C‐NMR (100 MHz, CDCl_3_) δ: 200.4, 174.5, 156.0, 79.6, 52.9, 46.1, 33.7, 32.7, 28.3, 27.0, 22.8 (Fig. S2).

#### Step 2

*N* ^α^‐Boc‐*N* ^ε^‐thioacetyl‐l‐lysine (2.75 g, 9 mmol) was dissolved in dry CH_2_Cl_2_ (25 mL), and trifluoroacetic acid (15 mL, 0.2 mol, 22 eq) was added dropwise to the reaction mixture over 20 min. The reaction mixture was stirred at room temperature for 4 h. The biphasic reaction mixture was separated, and the organic layer was washed with CH_2_Cl_2_ several times. The product was evaporated to dryness affording a white solid powder (1.65 g, 90%). Spectral data of the compound: ^1^H‐NMR (400 MHz, D_2_O) δ: 3.96 (m, 1H), 3.51 (m, 2H), 2.39 (s, 3H), 1.78–1.97 (m, 2H), 1.58–1.75 (m, 2H); 1.24–1.51 (m, 2H) (Fig. S3); ^13^C‐NMR (100 MHz, D_2_O) δ: 202.5, 172.6, 52.8, 45.6, 32.2, 29.3, 26.3, 21.5 (Fig. S4); ESI‐FTMS calcd for C_8_H_16_N_2_O_4_S (M + H) –205.1005, *m*/*z* found 205.1000 (Fig. S5).

### General molecular biology

Chemicals in this study were purchased from Sigma‐Aldrich (St Louis, MO, USA). *E. coli* TOP10 cells (Thermo Fisher Scientific, Waltham, MA, USA) were used for cloning and expression. Cloning experiments were performed by PCR and the DNA Assembly Kit (New England Biolabs, Ipswich, MA, USA). Point mutations were made by the QuikChange II mutagenesis kit (Agilent Technologies, Santa Clara, CA, USA).

#### Western blotting

Purified proteins were fractionated by SDS/PAGE and transferred onto a PVDF membrane. The membrane was incubated at room temperature with gentle shaking in TTBS (Tris Buffered Saline, with Tween 20, pH 8.0) and 5% BSA blocking buffer for 60 min. Horseradish peroxidase‐conjugated acetylated‐lysine (Ac‐K^2^‐100) rabbit antibody (Cell Signaling Technology, Beverly, MA, USA) was diluted 1 : 1000 and soaked the membrane overnight at 4 °C. The membrane was prepared for detection using Pierce™ ECL western blotting substrates (Thermo Fisher Scientific).

### The superfolder GFP readthrough assay

The assay follows a previous protocol 54. Strains harboring the superfolder GFP (sfGFP) reporter gene in the pBAD plasmid and the genes of AcKRS and TAcKRS variants and tRNA^Pyl^ in the pTech plasmid were inoculated into 2 mL LB medium. The overnight culture was diluted with fresh LB medium to an attenuance of 0.15 at 600 nm, supplemented with non‐canonical amino acids (ncAAs), 100 μg·mL^−1^ ampicillin and 50 μg·mL^−1^ chloramphenicol; 1 mm arabinose was added to induce the expression of sfGFP. Two hundred microliters of culture of each strain was transferred to a 96‐well plate. Cells were shaken for 12 h at 37 °C, with monitoring of fluorescence intensity (excitation 485 nm, emission 528 nm, bandwidths 20 nm) and attenuance (*D*_600_) by the microplate‐reader.

### AcKRS variant library construction and selection for TAcK‐specific variants

For variant library construction, three residues (F271, F313 and W382) of AcKRS were randomly mutagenized with three primers (AcKRS271‐QF: GCACCGAACCTGNNNAATTAT‐GCCCGTAAACTG; AcKRS313‐QF: CACCATGCTGAACTTTNNNCAAATGGGCTCG; and AcK RS382‐QF: GGTATTGACAAACCGNNNATCGGCGC G, NNN is completely randomization of corresponding positions) by the QuikChange multisite‐directed mutagenesis kit (Agilent Technologies). Selections were performed as described before 35. For the first round positive selection, 50 ng of pBK‐AcKRS (Km^r^) library plasmid was introduced into 50 μL of *E. coli* TOP10 (10^8^ cells) with the positive selection plasmid pCAT‐pylT (Tet^r^) that has a mutant *cat* gene with an amber stop codon at position 112 and tRNA^Pyl^. The transformants were recovered in 1 mL SOC (Super Optimal broth with Catabolite repression) at 37 °C for 2 h, and then cultivated in 100 mL LB‐TK (containing 10 μg·mL^−1^ tetracycline and 25 μg·mL^−1^ kanamycin) overnight at 37 °C. Of the overnight culture, 100 μL was inoculated into 5 mL fresh LB‐TK‐TAcK (LB‐TK containing 2 mm TAcK). After growing at 37 °C for 4 h, 200 μL culture was plated on LB‐TK‐TAcK‐Cm (LB‐TK‐TAcK containing 350 μg·mL^−1^ chloramphenicol) plates. Here we used 350 μg·mL^−1^ chloramphenicol because cells harboring the original AcKRS and tRNA^Pyl^ as well as the mutant *cat* gene could survival at 300 but not 350 μg·mL^−1^ chloramphenicol in the same condition (Fig. S6). The plates were incubated at 37 °C for 48 h, and all the colonies growing on the plates were scraped and resuspended in 10 mL LB‐TK‐TAcK. After additional incubation for 4 h at 37 °C, total plasmids were extracted, and the pBK‐AcKRS library plasmids were isolated by agarose gel electrophoresis and purified by the gel purification kit.

For the negative selection, 10 ng pBK‐AcKRS library plasmids from the positive selection were transformed into 50 μL *E. coli* TOP10 (10^8^ cells) with negative selection plasmid pAraCB2‐pylT (Cm^r^) that has tRNA^Pyl^ and a mutant *ccdB* gene with two amber stop codons at positions 13 and 44. The transformants were recovered in 2 mL SOC at 37 °C for 2 h, and then 50 μL was plated on LB‐CKara (containing 50 μg·mL^−1^ chloramphenicol, 25 μg·mL^−1^ kanamycin and 0.2% arabinose) plates. After incubation at 37 °C overnight, all the colonies were harvested, and pBK‐AcKRS library plasmids were isolated as described above for the next positive selection. One nanogram of pBK‐AcKRS library plasmids from the negative selection were introduced into 50 μL TOP10 (10^8^ cells) with the positive selection plasmid pCAT‐pylT. The transformants were recovered in 1 mL SOC at 37 °C for 2 h. It was transferred and cultivated in 50 mL LB‐TK overnight at 37 °C. Of the overnight culture, 100 μL was inoculated into 5 mL fresh LB‐TK‐TAcK. To obtain individual positive colonies at this stage, the cultures were diluted with fresh LB‐TK‐TAcK and plated on LB‐TK‐TAcK‐Cm plates. After 48 h of incubation, 69 colonies were selected, and each clone was inoculated into 1 mL LB‐TK and incubated overnight at 37 °C. Of the overnight culture, 100 μL was diluted with fresh 1 mL LB‐TK with or without 2 mm TAcK. After growing at 37 °C for 3 h, cell cultures were spotted on an LB‐TK‐TAcK‐Cm or LB‐TK‐Cm plate, separately. Finally, 17 clones were found to grow only on LB‐TK‐TAcK‐Cm plate. The pBK‐TAcKRS plasmid isolated and extracted from each clone was sent for DNA sequencing.

### Protein expression and purification

The procedure has small modifications from previous protocols 54, 55. The genes of target proteins were cloned into the pBAD plasmid with a C‐terminal His_6_‐tag, and transformed into Top10 cells together with the pTech plasmid harboring genes of tRNA^Pyl^ and TAcKRS for expression. The expression strain was grown on 400 mL LB medium supplemented with 100 μg·mL^−1^ ampicillin and 50 μg·mL^−1^ chloramphenicol at 37 °C to an attenuance of 0.6–0.8 at 600 nm, and protein expression was induced by the addition of 1 mm arabinose and supplemented with 5 mm TAcK. Cells were incubated at 30 °C for an additional 8 h, and harvested by centrifugation at 5000 ***g*** for 10 min at 4 °C. The cell paste was suspended in 15 mL of lysis buffer (50 mm Tris pH 7.5, 300 mm NaCl, 20 mm imidazole) with the protease inhibitor cocktail (Roche, Basel, Switzerland), and broken by sonication. The crude extract was centrifuged at 20 000 ***g*** for 25 min at 4 °C. The soluble fraction was filtered with a 0.45 μm filter and loaded onto a column containing 1 mL of Ni‐NTA resin (Qiagen, Hilden, Germany) previously equilibrated with 20 mL lysis buffer. The column was washed with 20 mL wash buffer (50 mm Tris pH 7.5, 300 mm NaCl, 50 mm imidazole), and eluted with 2 mL elution buffer (50 mm Tris pH 7.5, 300 mm NaCl, 150 mm imidazole). The elution fraction was desalted with desalting buffer (50 mm Tris pH 7.5, 20 mm NaCl) with a PD‐10 column (GE Healthcare Life Sciences, Pittsburgh, PA, USA).

### LC‐MS/MS analyses

The procedure has small modifications from the previous protocol 55. The purified proteins were trypsin digested by a standard in‐gel digestion protocol, and analyzed by LC‐MS/MS on an LTQ Orbitrap XL (Thermo Fisher Scientific) equipped with a nanoACQUITY UPLC system (Waters, Milford, MA, USA). A Symmetry C18 trap column (180 μm × 20 mm; Waters) and a nanoACQUITY UPLC column (1.7 μm, 100 μm × 250 mm, 35 °C) were used for peptide separation. Trapping was done at 15 μL·min^−1^, 99% buffer A (0.1% formic acid) for 1 min. Peptide separation was performed at 300 nL·min^−1^ with buffer A and buffer B (CH_3_CN containing 0.1% formic acid). The linear gradient was from 5% buffer B to 50% buffer B at 50 min, and to 85% B at 51 min. MS data were acquired in the Orbitrap with one microscan, and a maximum inject time of 900 ms followed by data‐dependent MS/MS acquisitions in the ion trap (through collision‐induced dissociation). The mascot search algorithm was used to search for the appropriate non‐canonical substitution (Matrix Science, Boston, MA, USA).

### Malate dehydrogenase activity assay

Malate dehydrogenase (MDH) activity assays were performed by following the instructions for the EnzyChrom^TM^ Malate Dehydrogenase Assay Kit (BioAssay Systems, Hayward, CA, USA). This non‐radioactive, colorimetric MDH assay is based on the reduction of the tetrazolium salt 3‐(4,5‐dimethylthiazol‐2‐yl)‐2,5‐diphenyltetrazolium bromide (MTT) in an NADH‐coupled enzymatic reaction to a reduced form of MTT that exhibits an absorption maximum at 565 nm. The increase in absorbance at 565 nm is proportional to the enzyme activity.

### CobB‐mediated deacetylation

The reaction was performed in buffer containing 40 mm HEPES (pH 7.0), 6 mm MgCl_2_, 1.0 mm NAD^+^, 1 mm DTT and 10% glycerol. Ten micrograms of MDH variants, 10 μg CobB and reaction buffer were incubated at 37 °C in a total volume of 100 μL. The treated proteins were used directly for western blotting.

## Results and Discussion

### Recognition of TAcK by acetyllysyl‐tRNA synthetase

**Figure 2:**
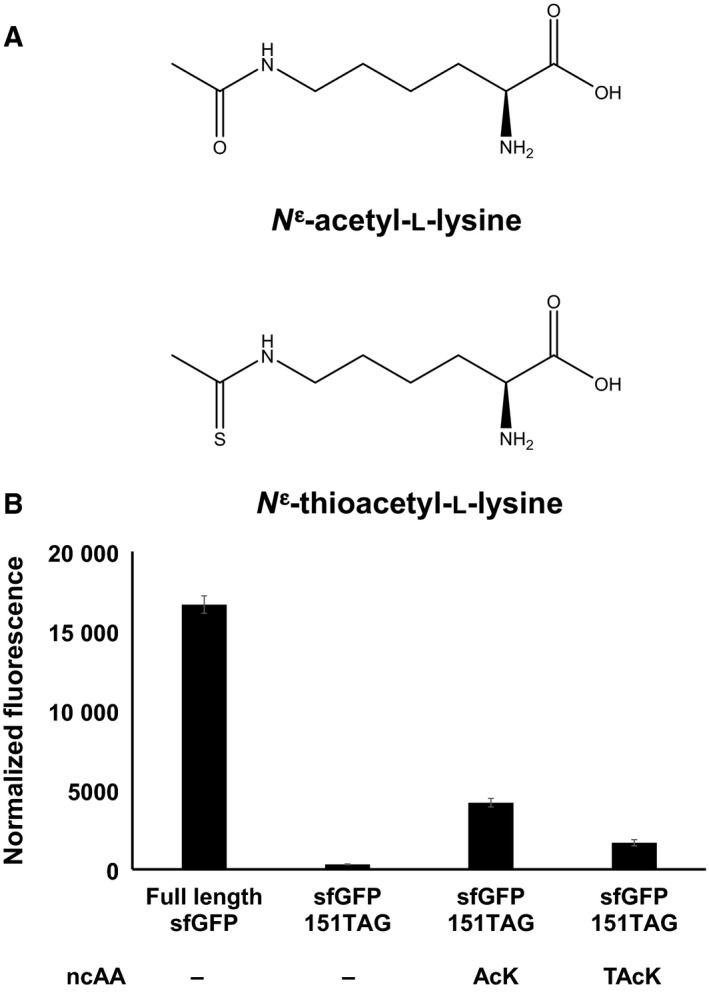
Recognition of TAcK by AcKRS. (A) The structures of AcK and TAcK. (B) The readthrough of the TAG codon at position 151 in sfGFP with AcK‐ or TAcK‐charged tRNA^P^ ^yl^ generated by AcKRS; 5 mm AcK or TAcK was used. Normalized fluorescence intensities were calculated from absolute fluorescence intensities read at 12 h normalized by corresponding cell densities. Mean values and standard errors were calculated from three replicates.

Due to structural similarity between AcK and TAcK (Fig. 2A) as well as the substrate flexibility of PylRS‐derived acetyllysyl‐tRNA synthetase (AcKRS) 56, 57, we firstly tested whether the original AcKRS could incorporate TAcK into proteins. Here we used the superfolder green fluorescent protein (sfGFP) as a reporter. The result showed that the suppression of the TAG codon at the permissive position 151 of sfGFP was only about 10% with the TAcK‐charged tRNA^Pyl^ that was generated by AcKRS (Fig. 2B). Thus we further engineered AcKRS for higher TAcK incorporation efficiency.

### Selection of TAcK‐specific aminoacyl‐tRNA synthetase variants

**Figure 3:**
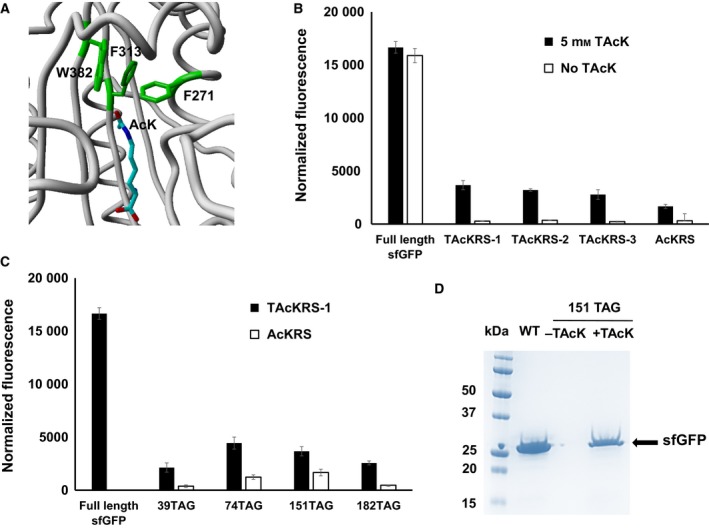
TAcKRS engineering. (A) The active site of AcKRS bound to AcK (PDB ID: 4q6 g). Residues F271, F313 and W382 were mutated in the complete randomization library. (B) The sfGFP readthrough assay for TAcKRS variants. Normalized fluorescence intensities were calculated from absolute fluorescence intensities read at 12 h normalized by corresponding cell densities. Mean values and standard errors were calculated from three replicates. (C) Comparison of TAcKRS‐1 and AcKRS efficiencies for TAcKRS incorporation at different positions in sfGFP; 5 mm TAcK was used in assays. The background of normalized fluorescence read from media without TAcK was subtracted for each reading. Mean values and standard errors were calculated from three replicates. (D) The Coomassie blue‐stained SDS/PAGE gel of purified full‐length sfGFP and its TAcK‐containing variant. The same volumes of elution fractions were loaded on the SDS/PAGE gel.

Based on the co‐crystal structure of the AcKRS and AcK (Fig. 3A), three residues of AcKRS (F271, F313 and W382) that are proximate to the oxygen atom of the acetyl‐group in AcK (replaced by a sulfur atom in TAcK) were selected for optimizing TAcK binding 56. A library of AcKRS variants with complete randomization of these three residues was generated and subjected to a series of chloramphenicol resistance‐based positive selections and a toxin CcdB‐based negative selection. After the second positive selection, we obtained three unique variants: TAcKRS‐1 (F271L and F313C), TAcKRS‐2 (F271L and F313M) and TAcKRS‐3 (F271N and F313I) (Table 1). The phenylalanine residue at position 271 or 313 was replaced by amino acids with smaller side chains to make a larger volume for the sulfur atom in TAcK. The tryptophan residue at position 382 was not changed in all the three variants, consistent with previous studies that have indicated that this tryptophan residue is important for the recognition of lysine analogs 35, 58, 59.

**Table 1:** Sequence comparison of TAcKRSs and AcKRS

WT PylRS	L266	L270	Y271	L274	C313	W382
AcKRS (ref. 37)	M	I	F	A	F	W
TAcKRS‐1 (this work)	M	I	L	A	C	W
TAcKRS‐2 (this work)	M	I	L	A	M	W
TAcKRS‐3 (this work)	M	I	N	A	I	W

Next, we used the sfGFP readthrough assay mentioned above to evaluate the TAcK incorporation efficiencies of these three variants. The best variant is TAcKRS‐1 (Fig. 3B). It is known that the ncAA incorporation efficiency depends on incorporation positions 60, and thus we tested the TAcK incorporation at different positions in sfGFP. The results showed that the engineered TAcKRS‐1 could increase TAcK incorporation up to 6‐fold from the original AcKRS (Fig. 3C). To lower the near cognate suppression of the TAG codon with canonical amino acids, TOP10 cells were used as the expression strain 61. We purified the TAcK‐containing sfGFP with a yield of 32 mg·L^−1^ culture (the yield of wild‐type sfGFP was 149 mg·L^−1^ culture in the same growth condition) (Fig. 3D), and the TAcK incorporation at the position 151 of sfGFP was confirmed with mass spectrometry (MS) (Fig. S7). The MS results also did not show any canonical amino acid incorporation at this position.

### Function of TAcK as a mimic of AcK

**Figure 4:**
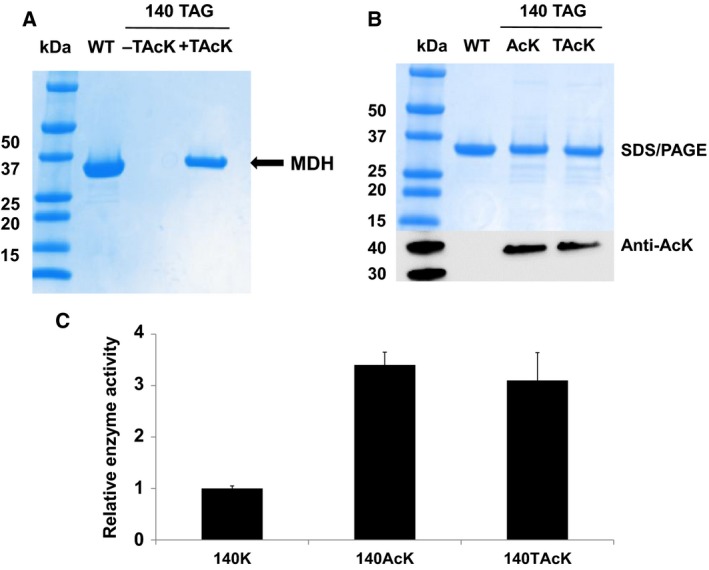
Characterization of TAcK‐containing MDH. (A) The Coomassie blue‐stained SDS/PAGE gel of purified full‐length MDH and its TAcK‐containing variant. The same volumes of elution fractions were loaded on the SDS/PAGE gel. (B) The Coomassie blue‐stained SDS/PAGE gel and western blotting of purified full‐length MDH and its AcK‐ and TAcK‐containing variants. The same amount of proteins were loaded on the gels. (C) The enzyme activities of MDH and its variants. Mean values and standard errors were calculated from three replicates. The enzyme activity of wild‐type MDH was set as 1.

To validate our system in functional proteins, we chose malate dehydrogenase (MDH), which plays a crucial role in the tricarboxylic acid cycle and glyoxylate bypass, as the target to characterize the replacement of lysine acetylation with thioacetylation. Our previous study showed that the acetylation of lysine residue 140 in MDH could increase the enzyme activity by 3.4‐fold 44, 54, and thus we mutated the corresponding position of K140 in the MDH gene to a TAG stop codon and expressed the MDH variant with the TAcK incorporation system. The yield of TAcK‐containing MDH was 9 mg·L^−1^ culture, while the yield of wide‐type MDH was 32 mg·L^−1^ culture in the same growth conditions (Fig. 4A). The incorporation of TAcK in MDH was confirmed by MS (Fig. S8). Also, MS analysis did not show any canonical amino acid incorporation at position 140 in MDH. Western blotting demonstrated that TAcK could also be detected by the anti‐AcK antibody with a similar intensity of AcK detection (Fig. 4B). The enzyme assay showed that the MDH‐140TAcK variant could increase the enzyme activity by 3‐fold, similar to that of the MDH‐140AcK variant (Fig. 4C). These results indicated that TAcK could serve as an ideal functional mimic of AcK.

### Deacetylation of TAcK by deacetylase

**Figure 5:**
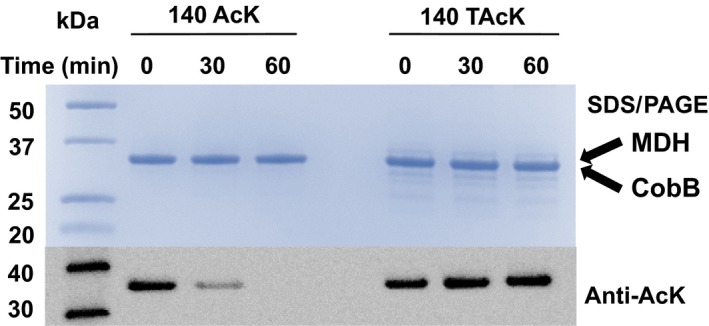
Resistance of TAcK‐containing MDH against CobB. The Coomassie blue‐stained SDS/PAGE gel and western blotting of CobB treatment of AcK‐ and TAcK‐containing MDH variants. The same amounts of proteins were loaded on the gels. CobB and MDH have similar molecular masses and were overlapped in the SDS/PAGE gel. Loaded samples were obtained at the start, 30 min and 60 min from deacetylation reaction mixtures.

To test resistance of TAcK‐containing proteins against deacetylases, we chose the CobB protein, which is a bacterial sirtuin deacetylase with a wide range of substrates as a representative 62, 63. We have shown that acetylation of K140 in MDH was sensitive to CobB 44. Since TAcK and AcK had a similar intensity against the anti‐AcK antibody (Fig. 3B), we performed western blotting to determine the deacetylation effect. After incubating the TAcK‐ or AcK‐containing MDH variant at position K140 with CobB individually, there was significant loss of acetylation of AcK‐containing MDH, while TAcK‐containing MDH could resist CobB deacetylase (Fig. 5).

## Conclusions

In summary, we established an *in vivo* genetic incorporation system of TAcK, which is both a good functional mimic and a non‐deacetylatable analog of AcK, and this will facilitate studies of protein acetylation in living bacterial cells. Furthermore, the pair of PylRS–tRNA^Pyl^ and its derivatives are orthogonal in eukaryotic cells, and previous studies have showed that AcKRS could incorporate AcK into protein by using mammalian cells as the hosts 36, so our approach could be further extended to living eukaryotic cells for a broader range of medical and pharmaceutical applications.

## Author contributions

MM and CF designed the experiments. SV, DTN, CG, QG and CF performed the experiments. SV, DTN, MM and CF analyzed the data and wrote the paper.

## Supporting information


**Fig. S1.** The ^1^H‐NMR spectrum of *N* ^α^‐Boc‐*N* ^ε^‐thioacetyl‐l‐lysine.
**Fig. S2.** The ^13^C‐NMR spectrum of *N* ^α^‐Boc‐*N* ^ε^‐thioacetyl‐l‐lysine.
**Fig. S3.** The ^1^H‐NMR spectrum of *N* ^ε^‐thioacetyl‐l‐lysine.
**Fig. S4.** The ^13^C‐NMR spectrum of *N* ^ε^‐thioacetyl‐l‐lysine.
**Fig. S5.** The ESI‐FTMS spectrum of *N* ^ε^‐thioacetyl‐l‐lysine.
**Fig. S6.** The growth of cells harboring the original AcKRS, tRNA^Pyl^ and a TAG‐containing mutant *cat* gene on different concentrations of chloramphenicol (Cm) in the LB‐TK‐TAcK plates.
**Fig. S7.** LC‐MS/MS analysis of sfGFP 151‐TAcK.
**Fig. S8.** LC‐MS/MS analysis of MDH 140‐TAcK.
