# Supplementary material for: Genetically encoding thioacetyl‐lysine as a non‐deacetylatable analog of lysine acetylation in Escherichia coli
Source: FEBS Open Bio. 2017 Oct 16;7(11):1805–14. doi: 10.1002/2211-5463.12320 (PMC5666399; doi:10.1002/2211-5463.12320)
Supplement: Supplementary file 1 — Fig. S1. The 1H‐NMR spectrum of N α‐Boc‐N ε‐thioacetyl‐l‐lysine. Fig. S2. The 13C‐NMR spectrum of N α‐Boc‐N ε‐thioacetyl‐l‐lysine. Fig. S3. The 1H‐NMR spectrum of N ε‐thioacetyl‐l‐lysine. Fig. S4. The 13C‐NMR spectrum of N ε‐thioacetyl‐l‐lysine. Fig. S5. The ESI‐FTMS spectrum of N ε‐thioacetyl‐l‐lysine. Fig. S6. The growth of cells harboring the original AcKRS, tRNAPyl and a TAG‐containing mutant cat gene on different concentrations of chloramphenicol (Cm) in the LB‐TK‐TAcK plates. Fig. S7. LC‐MS/MS analysis of sfGFP 151‐TAcK. Fig. S8. LC‐MS/MS analysis of MDH 140‐TAcK. [file FEB4-7-1805-s001.docx]

**Genetically encoding thioacetyl-lysine as a non-deacetylatable analog of lysine acetylation in *Escherichia coli***

Sumana Venkat, Dharma Theja Nannapaneni, Caroline Gregory, Qinglei Gan, Matt McIntosh and Chenguang Fan

**Supporting Information**

**Figure S1.** The ^1^H-NMR spectrum of *N*^α^-Boc-*N*^ε^-thioacetyl-l-lysine.


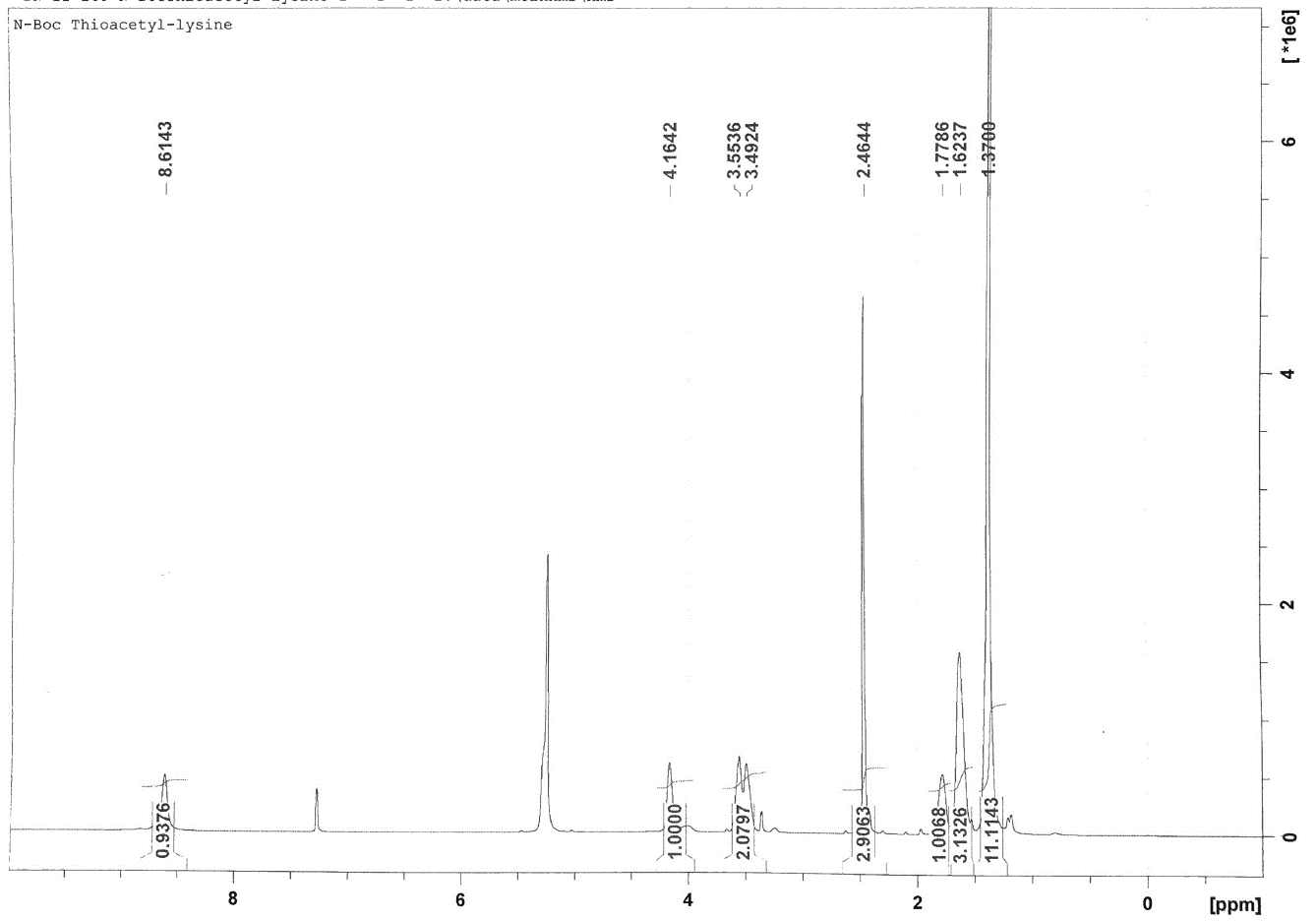


**Figure S2.** The ^13^C-NMR spectrum of *N*^α^-Boc-*N*^ε^-thioacetyl-l-lysine.

**Figure S3.** The ^1^H-NMR spectrum of *N*^ε^-thioacetyl-l-lysine.


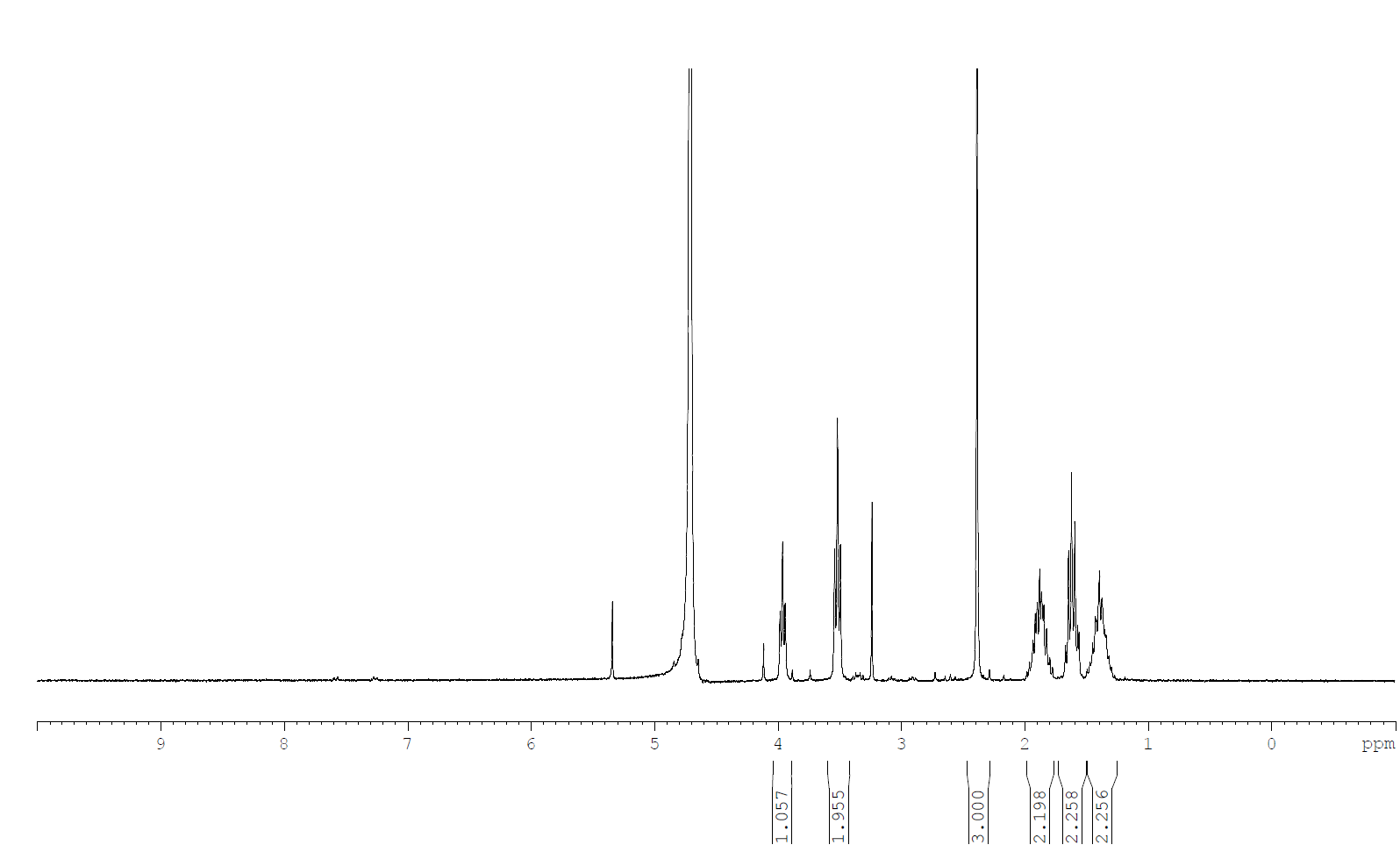


**Figure S4.** The ^13^C-NMR spectrum of *N*^ε^-thioacetyl-l-lysine.

**Figure S5.** The ESI-FTMS spectrum of *N*^ε^-thioacetyl-l-lysine.


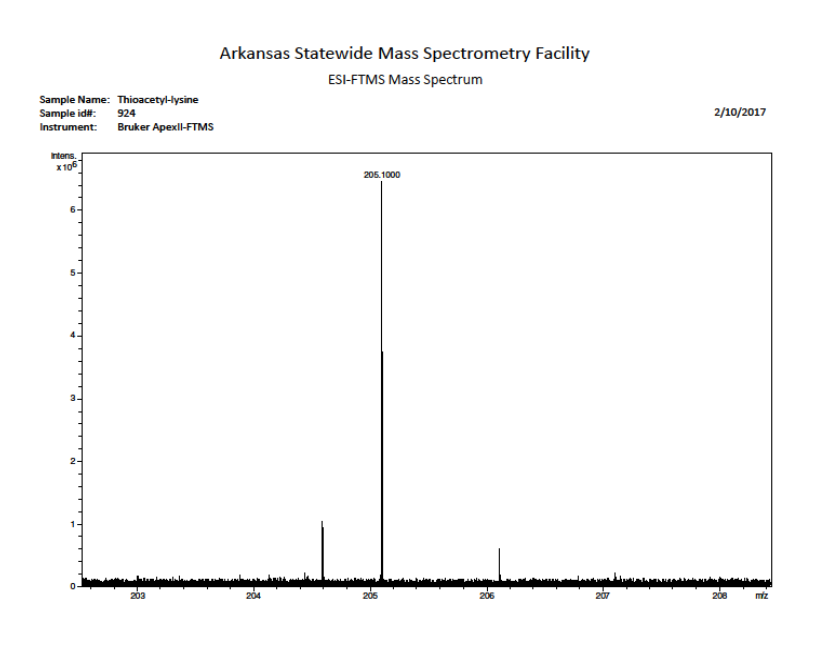


**Figure S6.** The growth of cells harboring the original AcKRS, tRNA^Pyl^, and a TAG-containing mutant *cat* gene on different concentrations of chloramphenicol (Cm) in the LB-TK-TAcK plates.

**
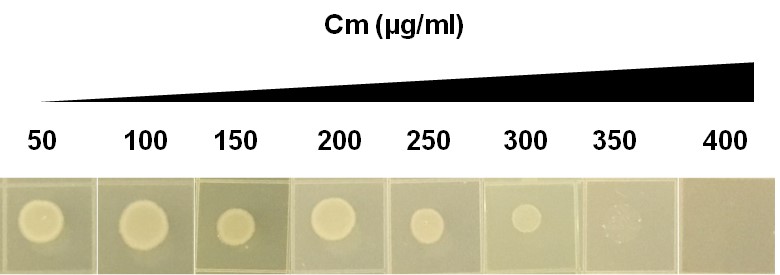
**

**Figure S7.** LC-MS/MS analysis of sfGFP 151-TAcK. The tandem mass spectrum of the peptide (residues 141–156) LEYNFNSHNVK^TH^ITADK from purified sfGFP 151-TAcK. K^TH^ denotes TAcK incorporation. The partial sequence of the peptide containing the TAcK can be read from the annotated b or y ion series. Matched peaks were in red.


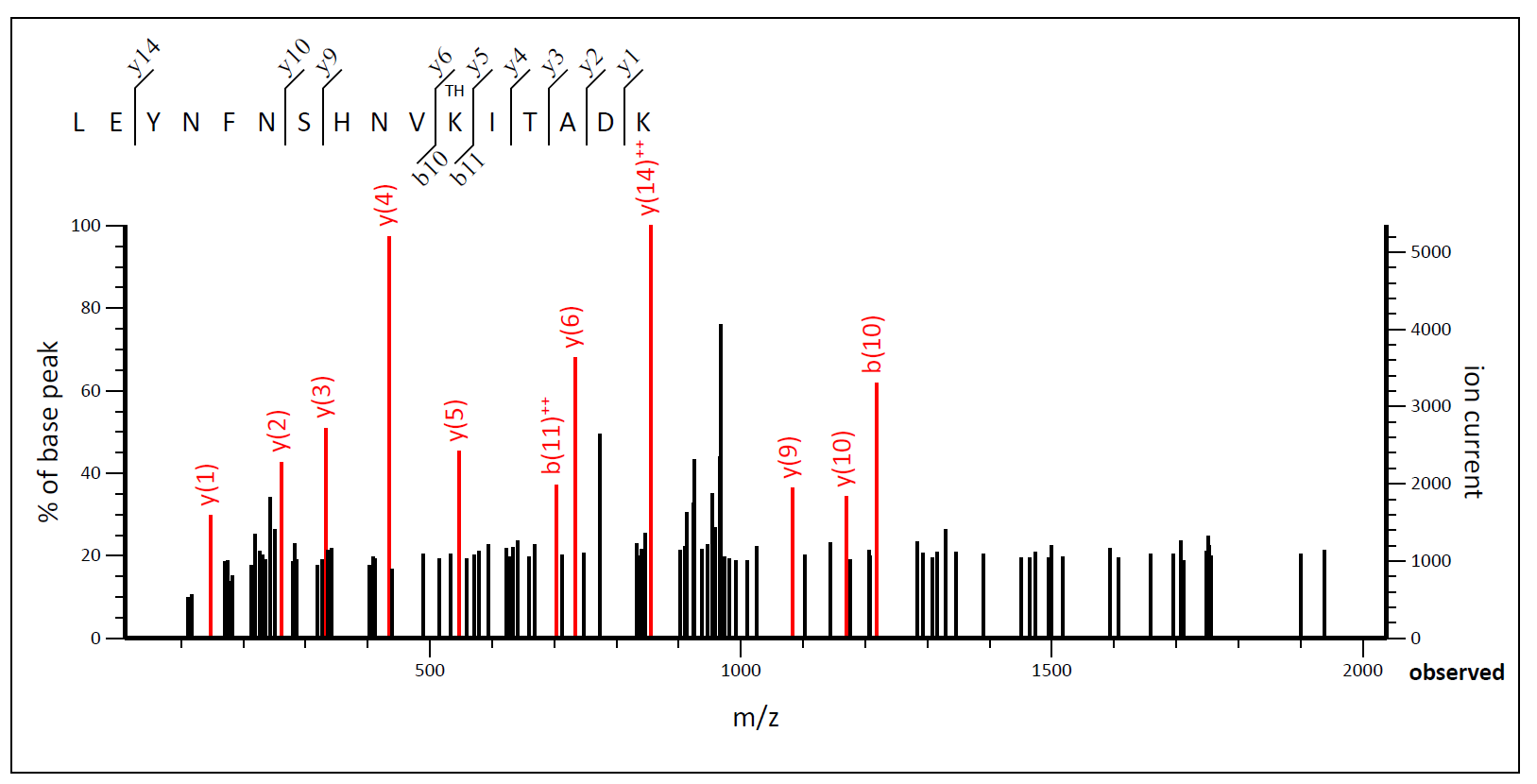


| **#** | **b** | **b++** | **Seq.** | **y** | **y++** | **#** |
| --- | --- | --- | --- | --- | --- | --- |
| **1** | 114.0913 | 57.5493 | **L** |  |  | **16** |
| **2** | 243.1339 | 122.0706 | **E** | 1837.8538 | 919.4305 | **15** |
| **3** | 406.1973 | 203.6023 | **Y** | 1708.8112 | **854.9092** | **14** |
| **4** | 520.2402 | 260.6237 | **N** | 1545.7478 | 773.3776 | **13** |
| **5** | 667.3086 | 334.1579 | **F** | 1431.7049 | 716.3561 | **12** |
| **6** | 781.3515 | 391.1794 | **N** | 1284.6365 | 642.8219 | **11** |
| **7** | 868.3836 | 434.6954 | **S** | ***1170.5936*** | 585.8004 | **10** |
| **8** | 1005.4425 | 503.2249 | **H** | ***1083.5615*** | 542.2844 | **9** |
| **9** | 1119.4854 | 560.2463 | **N** | 946.5026 | 473.7550 | **8** |
| **10** | **1218.5538** | 609.7805 | **V** | 832.4597 | 416.7335 | **7** |
| **11** | 1404.6365 | **702.8219** | **TAcK** | ***733.3913*** | 367.1993 | **6** |
| **12** | 1517.7206 | 759.3639 | **I** | ***547.3086*** | 274.1579 | **5** |
| **13** | 1618.7682 | 809.8878 | **T** | ***434.2245*** | 217.6159 | **4** |
| **14** | 1689.8054 | 845.4063 | **A** | ***333.1769*** | 167.0921 | **3** |
| **15** | 1804.8323 | 902.9198 | **D** | ***262.1397*** | 131.5735 | **2** |
| **16** |  |  | **K** | ***147.1128*** | 74.0600 | **1** |

**Figure S8.** LC-MS/MS analysis of MDH 140-TAcK. The tandem mass spectrum of the peptide (residues 134–142) KAGVYDK^TH^NK from purified MDH 140-TAcK. K^TH^ denotes TAcK incorporation. The partial sequence of the peptide containing the TAcK can be read from the annotated b or y ion series. Matched peaks were in red.


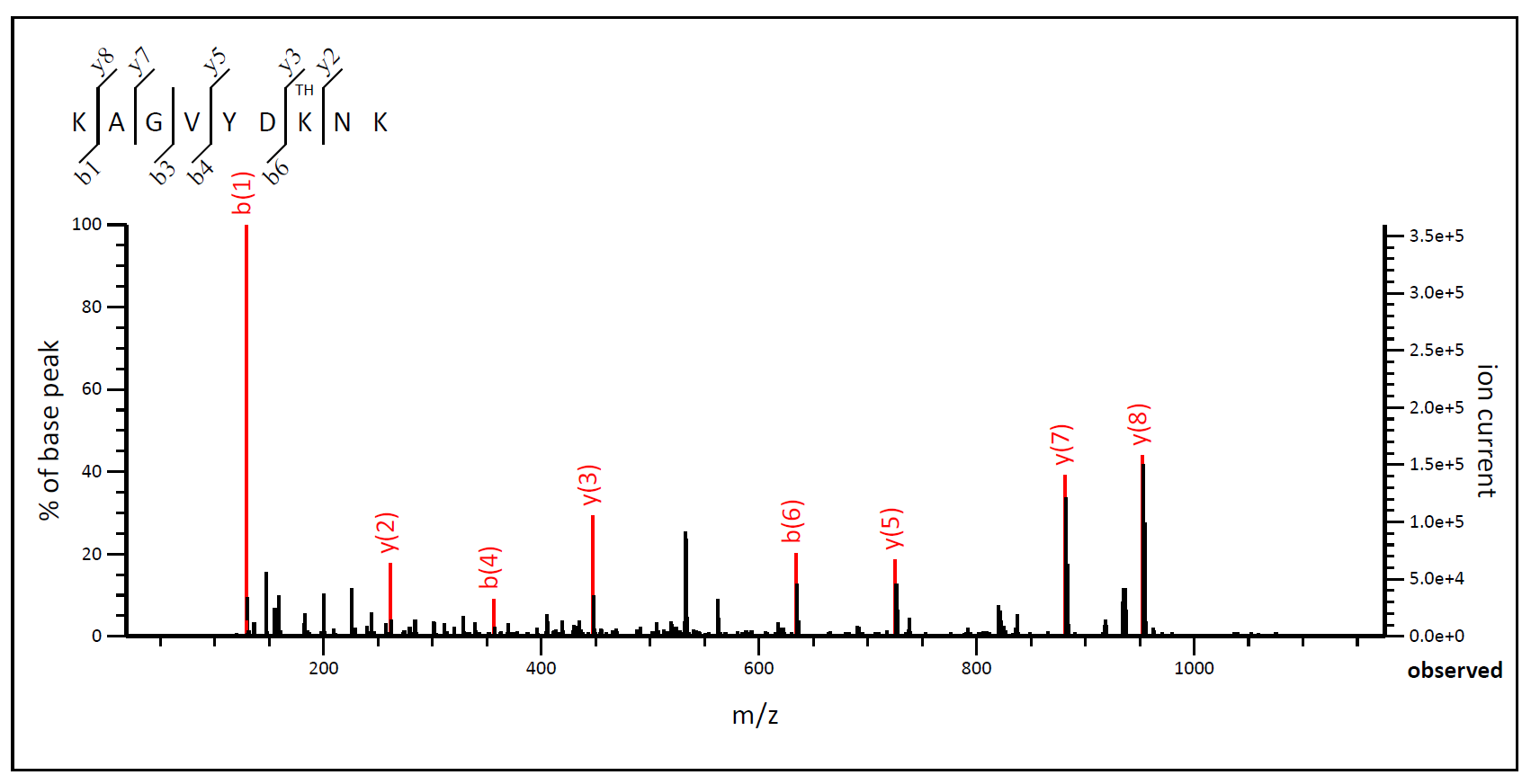


| **#** | **b** | **b++** | **Seq.** | **y** | **y++** | **#** |
| --- | --- | --- | --- | --- | --- | --- |
| **1** | **129.1022** | 65.0548 | **K** |  |  | **9** |
| **2** | 200.1394 | 100.5733 | **A** | ***952.4557*** | 476.7315 | **8** |
| **3** | 257.1608 | 129.0840 | **G** | ***881.4186*** | 441.2129 | **7** |
| **4** | **356.2292** | 178.6183 | **V** | 824.3971 | 412.7022 | **6** |
| **5** | 519.2926 | 260.1499 | **Y** | ***725.3287*** | 363.1680 | **5** |
| **6** | **634.3195** | 317.6634 | **D** | 562.2654 | 281.6363 | **4** |
| **7** | 820.4022 | 410.7047 | **TAcK** | ***447.2384*** | 224.1228 | **3** |
| **8** | 934.4451 | 467.7262 | **N** | ***261.1557*** | 131.0815 | **2** |
| **9** | 1119.4854 | 560.2463 | **K** | 147.1128 | 74.0600 | **1** |
